# Influence of benign prostatic hyperplasia patterns detected with MRI on the clinical outcome after prostatic artery embolization

**DOI:** 10.1186/s42155-023-00357-y

**Published:** 2023-03-02

**Authors:** Matthias Boschheidgen, Rouvier Al-Monajjed, Peter Minko, Kai Jannusch, Tim Ullrich, Karl Ludger Radke, Rene Michalski, Jan Philipp Radtke, Peter Albers, Gerald Antoch, Lars Schimmöller

**Affiliations:** 1 Medical Faculty, Department of Diagnostic and Interventional Radiology, University Dusseldorf, Moorenstr. 5, Dusseldorf, D-40225 Germany; 2 Medical Faculty, Department of Urology, University Dusseldorf, Moorenstr. 5, Dusseldorf, D-40225 Germany

**Keywords:** Prostatic arterial embolization, Benign prostatic hyperplasia, Hyperplasia patterns, Prostate MRI

## Abstract

**Background:**

To investigate the influence of benign prostatic hyperplasia (BPH) patterns detected with MRI on clinical outcomes after prostatic artery embolization (PAE).

**Materials & methods:**

This retrospective study included 71 consecutive patients with lower urinary tract symptoms (LUTS), who underwent magnetic resonance imaging (MRI) of the prostate followed by PAE at a single centre. MRI scans were evaluated and BPH patterns were determined according to Wasserman type and a modified BPH classification. Additionally, scans were evaluated regarding the presence of adenomatous-dominant benign prostatic hyperplasia (AdBPH). LUTS were assessed using the International Prostate Symptom Score (IPSS) and urinary flow rate (Qmax). Follow-up examination included MRI and clinical outcome.

**Results:**

For clinical outcome at follow-up, IPSS showed median reduction of 54% (IQR 41—75%) and Qmax improved by 4.1 ml/s. We noted significant reduction in volume, intraprostatic protrusion, and prostatic urethral angle in our collective (*p* < 0.01). Median volume reduction was 25% (IQR 15%—34%). Bilateral embolization was a significant predictor for volume reduction at follow-up. Multiple linear regression analysis showed significant effect of high initial volume on reduction in IPSS after treatment (*p* < 0.01). Presence of AdBPH was significantly associated with both, volume loss and clinical improvement in terms of IPSS reduction (*p* < 0.01). Neither BPH pattern based on the Wassermann type nor modified BPH classification were significantly related with postinterventional IPSS and volume loss.

**Conclusions:**

Men benefit from PAE regardless the macroscopic BPH MRI pattern. Preinterventional prostate volume and presence of AdBPH on MRI should be considered for outcome prognosis after PAE.

## Background

Prostatic artery embolization (PAE) is an established non-invasive therapeutic option for treatment of symptomatic benign prostatic hyperplasia (BPH) (Abt et al. 2018; Bilhim et al. 2022; Carnevale et al. 2016; Kovács et al. 2020; McWilliams et al. 2019; Pisco et al. 2013a; Sun et al. 2020). Nevertheless, there is still uncertainty about possible predictors to estimate the clinical outcome after therapy (Abt et al. 2019; Assis et al. 2015, 2017; Bilhim et al. 2016; Guneyli et al. 2016; Hacking et al. 2019). As a preinterventional factor, baseline prostate volume could have an impact on both, size reduction as well as clinical improvement (Abt et al. 2019; Maclean et al. 2020; Pisco et al. 2013b, 2016; Xiang et al. 2021). However, Bagla et al. did not report on this relationship (Bagla et al. 2015). Little et al. used a different approach, focussing on the pathological composition at preinterventional magnetic resonance imaging (MRI), defining the presence of adenomatous-dominant benign prostatic hyperplasia as a prognostic factor. These adenomas tend to be more perfused, also seen as a strong enhancement in contrast enhanced sequences, hence leading towards a better response on endovascular treatment with PAE (Little et al. 2017). MRI findings after PAE contain primarily infarction, inducing a decline in prostate volume (Frenk et al. 2014; Kisilevzky and Faintuch 2016). Still, the extent of volume loss showed no clear correlation with clinical outcome, e.g., International Prostate Symptom Score (IPSS) or maximum uroflow rate (Qmax). Other outcome measures as intravesical prostatic protrusion (IPP) may be influenced by PAE, however, the role as a predictor in terms of clinical benefit is arguable (Lin et al. 2016).

A couple of years ago, Wasserman et al. proposed a MRI-based classification on different patterns of BPH, considering the localization of hyperplastic nodules (Wasserman et al. 2015). They stated the presence of six different patterns with an additional pattern for prostates, who do not fit into one of the other groups. In our experience, the predominant patterns are either pedunculated with bilateral transition zone (TZ) and retrourethral enlargement (pattern 5), bilateral TZ and retrourethral enlargement (pattern 3) or bilateral TZ enlargement (pattern 1).

There are only a few study groups who implemented the classification to their research. Grivas et al. investigated on the effect of BPH pattern in a collective diagnosed with prostate cancer receiving prostate MRI followed by radical prostatectomy (Grivas et al. 2017). Follow-up examination included clinical outcome regarding lower urinary tract symptoms (LUTS) and continence. Although the indication for operation was different from the typical collective with LUTS due to BPH, it is an interesting approach. A recently published German review gives insight into the use of MRI apart from cancer imaging, containing important additional information on the structure and form of BPH (Oerther et al. 2022).

The need for a minimally invasive therapeutic option is obvious. However, the right selection of patients remains difficult as defined outcome parameters showed inconsistent results in previous studies (Abt et al. 2019; Assis et al. 2017; Bagla et al. 2015). The aim of the study is to corelate the BPH pattern on MRI with the outcome of PAE in terms of volume reduction as well as clinical improvement to ameliorate patient selection.

## Materials & methods

### Study design

This retrospective study was approved by the local ethics committee. Between October 2019 and December 2021, consecutive patients who received PAE and prior MRI were included in this study. All patients had severe LUTS due to BPH, refractory to medical treatment, and had been seen by an experienced urologist. Decisions for PAE were made in consensus between patients, urologists, and radiologists. Patients were informed about the intervention at least 24 h before treatment and written informed consent was present from all patients. Prostate cancer was excluded prior to PAE by MRI and biopsy (in case of suspicious MRI or clinical indication). All interventions were performed by an interventional radiologist with 4 years of experience in PAE (L.S.).

Pre-interventional MR scans were analysed retrospectively. The Prostate Imaging Reporting and Data System (PIRADS) classification, prostate volume, intravesical prostatic protrusion (IPP), prostatic urethral angle (PUA), presence of adenomatous-dominant benign prostatic hyperplasia (AdBPH), and pattern of hyperplasia according to the Wasserman classification and modified BPH classification with only three different patterns, classified by the predominance of the distribution of hyperplasia (preurethral, retrourethral, biurethral) were assessed (Table 1). The first three Wasserman patterns correspond to the modified patterns, Wasserman 4 and 6 were classified as modified pattern 2, and Wasserman 5 was allocated into modified pattern 3. Urological examination took place before the intervention. Current BPH medication, presence of urine catheter, IPSS, and Qmax were defined as clinical variables. PAE was defined as technically successful, if both sides were embolized, partially successful, if only one side was embolized, and unsuccessful, if embolization of the prostatic arteries failed. Patients received follow-up MRI without the application of contrast media and urological examination three months and one year after embolization. Outcome parameters in MRI included prostate volume, IPP, and PUA. IPSS, BPH medication, and presence of bladder catheter were defined as clinical outcome parameters. Finally, MRI and clinical parameters were compared pre- and post-embolization.

**Table 1:** MRI classification of BPH subtypes

**Wasserman**^a^	**Modified BPH classification**
Type 1: bilateral TZ	1: preurethral
Type 2: retrourethral	2: retrourethral
Type 3: bilateral TZ and retrourethral	3: biurethral
Type 4: solitary pedunculated	
Type 5: bilateral TZ and pedunculated	
Type 6: subtrigonal	

*TZ* Transition zone,  *BPH* Benign prostate hyperplasia

^a^ Wasserman et al. AJR 2015; 205(3): 564–571. https://doi.org/10.2214/AJR.14.13602

### Imaging acquisition

All MRI scans were conducted on 3 T MRI scanners (Magnetom Prisma; Siemens Healthineers, Forchheim, Germany) using a 60-channel phased-array surface coil. MRI parameters were chosen according to international recommendations for prostate MRI and contained T2-weighted turbo spin echo (TSE) sequences in 3 planes (T2WI), diffusion-weighted imaging (DWI), and T1-weigehted imaging (Franiel et al. 2021). If there was no suspicion for cancer after the acquisition of non-enhanced images, we acquired a MRA with intravenous contrast media (3D T1 FLASH; TE 1.24, TR 3.7 ms, Slice thickness 0.9 mm, FOV 350 mm, 20 ml CM dose, 2 ml/s injection rate). The field of view was placed over the lower abdominal aorta and iliac vessels and involved PA and entire prostate tissue. For follow-up MRI, we acquired T2-weighted turbo spin echo (TSE) sequences in three planes (T2WI), diffusion-weighted imaging (DWI) and T1-weighted imaging. Prostate volume was measured by software volumetric analysis (DynaCAD, Philips Healthcare).

### Prostatic artery embolization (PAE)

 All PAE were performed using a therapeutic angiographic unit with a digital flat-panel detector system (Allura Xper FD20; Phillips Healthcare, Best, The Netherlands) equipped with cone beam CT option. First, the right common femoral artery (CFA) was punctured in seldinger technique and 5F-sheath was inserted. Probing of left internal iliac artery was conducted using a 5F-RIM, 5F-SIM-1, and a hydrophilic guidewire. Next, DSA in an angulated series (LAO 30°, CRAN 10°) or CBCT (using 3D road map) was performed to identify the origin of the left prostatic artery (PA). Afterwards, a microcatheter (Direxion, Bern-Shape, 2.7/2.4 Fr; Boston Scientific; Marlborough, MA, USA) was coaxially inserted and probing of left the PA was performed using a microwire (Fathom 0.016’’). CBCT was executed applying 5 ml of diluted contrast (Iomeron 400/NaCl; 50:50) at 0.2 ml/s to check embolization position and exclude collateral vessels. If collaterals were observed to penis, bladder or rectum, these branches were occluded temporarily using Gelfoam. Microcatheter was placed distally in wedge position. Embolization was conducted using 250 µm-particles (Embozene Microspheres, Varian Medical Systems, Paolo Alto, CA) and subsequent 350-500 µm-Contour-particles (Boston Scientific, Natick, Massachusetts) until full stasis in the vessel was achieved. Embolization was performed subsequently on the right side in the same way. In case of insufficient probing/catheter positioning (e.g., due to vessel stenosis) or if protective embolization of collateral vessels was unfeasible on one or both sides, prostate embolization was not conducted, respectively. After completing embolization all extraneous material was eliminated, and the puncture side was closed using 6F Angioseal.

### Statistical analysis

Statistics were performed using IBM SPSS® Statistics (Version 27, IBM Corp). *P*-values < 0.05 were defined as statistically significant. Descriptive statistics included mean, median, standard deviation, and interquartile ranges. Wilcoxon signed rank test was performed to check for statistically significant differences in outcome parameters between baseline and follow-up. For the prediction of volume change and IPSS reduction due to treatment, multiple linear regression was performed including different input parameters (age, successful embolization, volume, IPP, PUA, AdBPH, IPSS, urinary retention, type of hyperplasia). The predictive value of the resulting model with nine predictors was assessed by reporting the adjusted r^2^ value. For comparison of outcome for different patterns of hyperplasia and for different volume groups, Kruskal–Wallis test was performed and, and to compare results depending on the presence of AdBPH and the effect of bilateral embolization, Wilcoxon rank sum test was conducted.

## Results

### Patients

Baseline characteristics of all seventy-one patients are shown in (Table 2). PIRADS classification of 3 was present in three patients while PIRADS classification of 4 or 5 was present in none of the patients. PI-RADS 3 patients received biopsy prior to embolization to exclude prostate cancer. In the rest of the cohort, MRI revealed PIRADS classification of 1 or 2. Prostate volume differed widely in this collective, the smallest prostate measured 30 ml while the largest prostate had a volume of 306 ml. Bilateral embolization was performed in 61/71 patients whereas unilateral embolization was conducted in 8/71 patients. In 3% of the patients, intervention failed due to technical challenging vessel anatomy and failure of catheterization.

**Table 2:** Baseline parameters

**Patients** number; n	71
**Age** years; median (IQR)	72 (67—78)
**BMI** kg/m^2^; median (IQR)	25 (24—28)
**PSA** ng/ml; median (IQR)	4.4 (2.7—8.8)
**PSAD** ng/ml/cm^3^; median (IQR)	0.05 (0.03—0.08)
**PI-RADS category**	2 (2 – 2)
**Technical success** n	Both sides = 61 One side = 8 None = 2

*BMI* Body mass index, *PSA* Prostate specific antigen, *PSAD* PSA density

### Influence of MRI BPH pattern

**Fig. 1:**
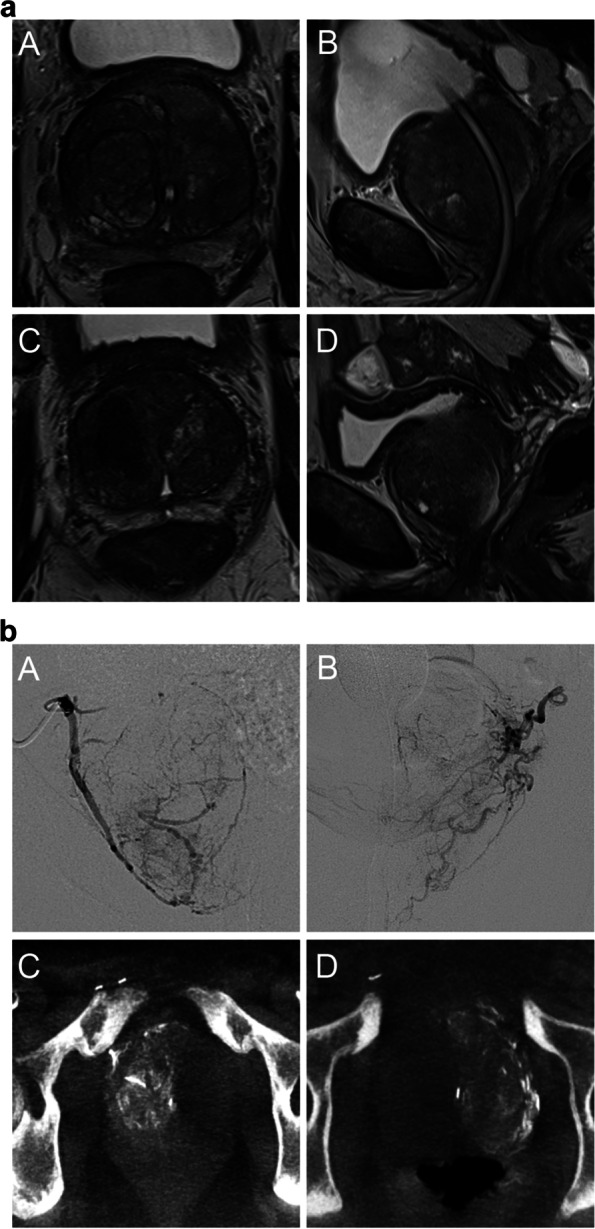
**a** Wasserman type 1, modified BPH classification 1 (preurethral); A: T2-weighted axial baseline B: T2-weighted sagittal baseline; compression and displacement of the urethra posteriorly; C: T2-weighted axial follow-up D: T2-weighted sagittal follow-up. Volume reduction of 130 ml to 76 ml (42%); IPSS 10 to 2 (80%). **b** A/B: Digital subtraction angiography from right/left prostatic artery; C/D cone-beam-computed tomography with contrast injection from right/left prostatic artery

**Fig. 2:**
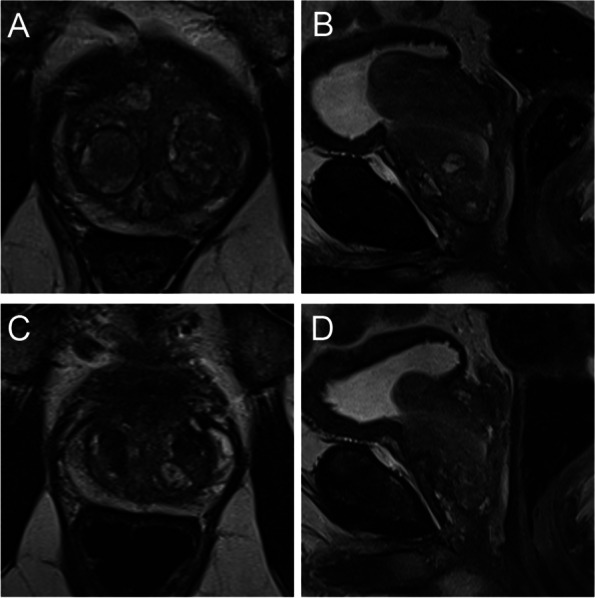
Wasserman type 2, modified BPH classification 2 (retrourethral); **A** T2-weighted axial baseline **B** T2-weighted sagittal baseline; retrourethral dominant hyperplasia with pedunculated nodules; **C** T2-weighted axial follow-up **D** T2-weighted sagittal follow-up. Volume reduction of 95 ml to 51 ml (46%); IPSS 12 to 6 (50%)

**Fig. 3:**
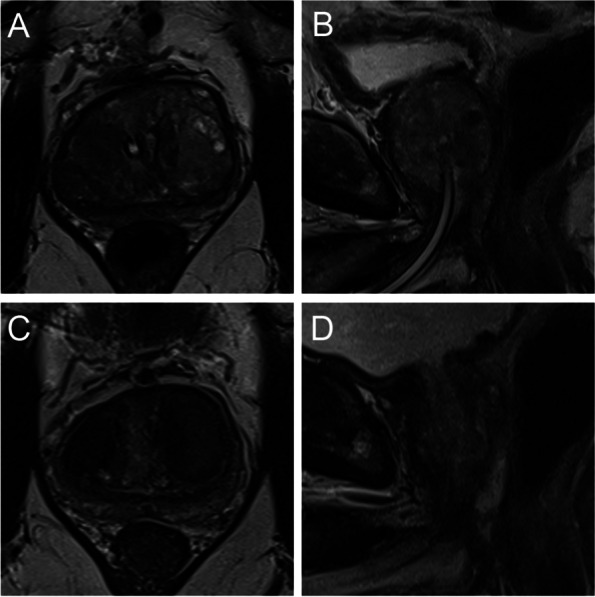
Wasserman type 3, modified BPH classification 3 (biurethral); **A** T2-weighted axial baseline **B** T2-weighted sagittal baseline; preurethral dominant hyperplasia without pedunculation; **C** T2-weighted axial follow-up **D** T2-weighted sagittal follow-up. Volume reduction of 76 ml to 36 ml (53%); IPSS 24 to 10 (58%)

**Fig. 4:**
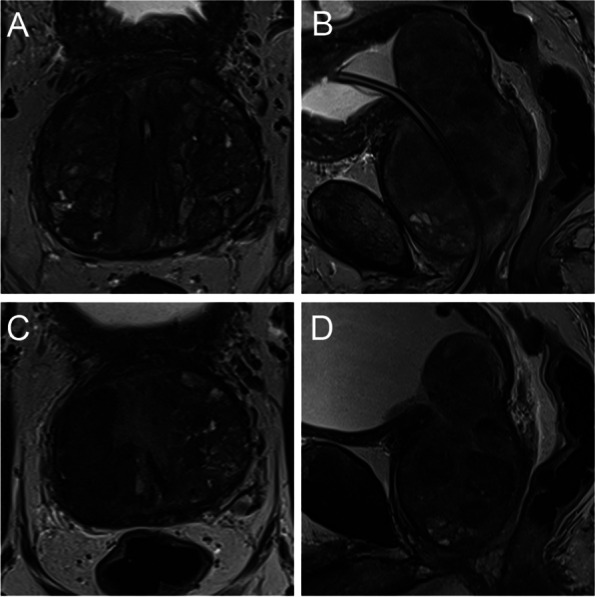
Wasserman type 5, modified BPH classification 3 (biurethral); **A** T2-weighted axial baseline **B** T2-weighted sagittal baseline; bilateral TZ hyperplasia with dominantly pedunculated pattern; **C** T2-weighted axial follow-up **D** T2-weighted sagittal follow-up. Volume reduction of 293 ml to 175 ml (40%); IPSS 17 to 2 (88%)

For comparison of groups with different BPH patterns, Kruskal–Wallis-test was performed (Table 3). Distribution among Wasserman type was imbalanced, as only one patient with type 4 and five patients with type 2 were included. Neither BPH patterns based on the Wasserman type nor modified BPH classification were significantly associated with postoperative LUTS as measured by IPSS during follow-up (Figs. 1, 2, 3 and 4). Furthermore, both groups of different individual BPH patterns (Wasserman type and modified BPH classification) showed no significant differences in volume change after PAE (Table 3).

**Table 3:** Comparison of changes in volume and IPSS for different patterns of hyperplasia, presence of AdBPH, technical success of embolization, and different volume groups

	**IPSS reduction**		**Volume reduction**	
	**mean**	**SD**	**mean**	**SD**
**BPH type according to Wasserman**				
** type 1 (*n* = 18)**	56%	33%	25%	11%
** type 2 (*n* = 5)**	45%	34%	26%	10%
** type 3 (*n* = 15)**	54%	24%	22%	14%
** type 4 (*n* = 1)**	45%	na	28%	na
** type 5 (*n* = 17)**	38%	56%	25%	13%
* p*-value*	0.60		0.88	
**simplified BPH classification**				
** preurethral (*n* = 27)**	52%	33%	25%	11%
** retrourethral (*n* = 9)**	50%	29%	29%	10%
** biurethral (*n* = 30)**	45%	49%	22%	14%
* p*-value*	0.59		0.88	
**PAE unilateral vs. bilateral**				
** Unilateral (*n* = 7)**	21%	85%	14%	14%
** Bilateral (*n* = 59)**	52%	29%	26%	12%
* p*-value**	** < 0.01**		** < 0.01**	
**presence of AdBPH**				
** non-AdBPH (*n* = 13)**	36%	38%	21%	13%
** AdBPH (*n* = 53)**	51%	39%	25%	9%
* p*-value**	** < 0.01**		** < 0.01**	
**volume groups**				
< **100 ml (*n* = 43)**	43%	43%	23%	12%
** > 100 ml (*n* = 23)**	59%	27%	26%	13%
* p*-value**	** < 0.01**		** < 0.04**	

*AdBPH* Adenomatous-dominant benign prostatic hyperplasia, *BPH* Benign prostatic hyperplasia, *IPSS* International prostatic symptoms score, *SD* standard deviation, *PAE* prostatic artery embolization

^*^Kruskall-Wallis-test was used to check for statistical significance

^**^Wilcoxon-rank-sum-test was used to check for statistical significance

### Effect of PAE on clinical and MRI parameter

Follow-up-data was present for 66/69 patients, where at least unilateral embolization was achieved (Table 4). 63/69 patients had follow up at 3 months whereas 44/69 patients were examined at 12 months after intervention (mean follow-up interval 9 ± 6 months). In patients were data was available for both timepoints, only the one-year follow up was incorporated. We observed significant improvement in both clinical and imaging outcome parameters, although results varied widely. For IPSS scores, we had median reduction of 54% (IQR 41%—75%), although four patients showed worsening of clinical symptoms at follow-up. Considering Qmax, we could as well detect improvement in flow with a difference of 4.1 ml/s between baseline and follow-up. Focusing on imaging parameters in prostate MRI before and after intervention, we noted significant reduction in volume, IPP and PUA in our collective. Median volume reduction was 25% (IQR 15%—34%); in one patient we calculated higher prostate volume at follow-up 3 months after PAE.

**Table 4:** Outcome parameters initially and at follow up

		**Initial**	**Follow-up**	*P*-value*
**Number**	*n*	69	66	
**Volume**; ml	*Mean* ± *SD*	105 ± 60	79 ± 43	** < 0.01**
**IPP**; mm	*Mean* ± *SD*	13 ± 7	10 ± 9	** < 0.01**
**PUA**; degree	*Mean* ± *SD*	69 ± 15	60 ± 15	** < 0.01**
**Medication **number of drugs	*median (IQR))*	2 (0 – 3)	0 (0 – 3)	** < 0.01**
**IPSS**	*median (IQR)*	20 (15 – 24)	8 (4 – 13)	** < 0.01**
**Bladder catheter**		20	2	
**Qmax**; ml/s	*Mean* ± *SD*	11 ± 6	15 ± 9	**0.02**

*IPP* Intraprostatic protrusion, *PUA* Prostatic urethral angle, *IPSS* International prostatic symptoms score, *IQR* Interquartile range

^*^Wilcoxon-rank-sum-test was used to check for statistical significance

Multiple linear regression analysis for single parameters to predict relative changes in IPSS and prostate volume are shown in (Table 5). High initial volume was significantly related to a reduction in IPSS scores after treatment (*p*** < **0.01). When opposing patients with initial volume > 100 ml (23 patients) against patients with initial volume < 100 ml (43 patients), the group with higher volume had significantly greater reduction of IPSS (*p* < 0.01) (Table 3).

**Table 5:** Multiple regression analysis for the prediction of relative volume loss and relative IPSS reduction after PAE

	**IPSS** post PAE		**Prostate volume** post PAE	
	**r**	**p**	**r**	**p**
**Age**	-1.2	0.10	0.25	0.22
**Unilateral vs. bilateral PAE**	23.8	0.18	10.3	**0.03**
**Prostate volume;** initial	0.37	**0.01**	0.02	0.58
**IPP**	-1.23	0.25	0.27	0.33
**PUA**	-0.44	0.33	-0.18	0.12
**AdBPH**	-0.36	0.33	5.6	0.19
**BPH pattern**	3.16	0.61	-2.9	0.10
**IPSS;** initial	-0.28	0.73	0.02	0.94
**indwelling bladder catheter**	-31.1	0.07	7.8	0.07

*IPP* Intraprostatic protrusion, *PUA* Prostatic urethral angle, *AdBPH* Adenomatous-dominant benign prostatic hyperplasia, *BPH* Benign prostatic hyperplasia, *IPSS* International prostatic symptoms score, *PAE* prostatic artery embolization

For predicting volume loss after intervention, unilateral/bilateral PAE was the only significant parameter (*p* = 0.03). Wilcoxon-rank-sum-test revealed the same results with a mean (SD) reduction of 14% (14%) for unilateral embolization vs. 26% (12%) for bilateral embolization.

Looking at interior tissue composition of the prostate, presence of AdBPH, which was the case in 53/66 patients, was significantly associated with both volume loss and clinical improvement in terms of IPSS reduction (*p* < 0.01) when conducting Wilcoxon rank sum test (Table 3). Multiple linear regression analysis did not reveal a significant role as a predictor for outcome measures.

## Discussion

Appropriate patient selection for PAE is crucial, but there is a lack on reliable predictors for the clinical outcome after PAE. It is still a matter of discussion if volume reduction correlates with the clinical improvement in terms of a reduction in LUTS measured by different scores (Abt et al. 2019; Bilhim 2019; Carnevale et al. 2020; Frenk et al. 2014; Maclean et al. 2018, 2020). This study showed that all MRI BPH pattern had similar outcome in IPSS and volume reduction. However, significant outcome improvement was related to presence of AdBPH, larger prostate glands, and higher initial IPSS.

In our collective PAE was a safe and effective treatment for patients suffering from LUTS caused by BPH. We observed a significant reduction of prostate volume, IPP and PUA in follow-up MRI. More important, the effect on clinical improvement (IPSS reduction, no more need for medication) and volume reduction does not depend on the MRI BPH pattern identified in preinterventional scans. We investigated these outcomes for both, Wassermann MRI patterns and a simplified classification to look for any differences at follow-up. As we were not able to identify any discrepancies, a possible explanation is that macroscopic appearance plays no major role for selection of patients suitable for PAE. This stands in line with previously published results (Bilhim 2019; Maron et al. 2020; Meira et al. 2021; Xu et al. 2022; Yu et al. 2019). Adequate treatment for patients with dominant median lobe hyperplasia has been discussed controversially in the last couple of years. Median lobe hyperplasia causes IPP into the urinary bladder, causing in a ball-type obstruction of the bladder neck, increasing the urethral resistance during micturition (Chia et al. 2003). These patients tend to have lower urinary flow rates, higher risk of clinical progression of BPH, and show poor response to medical treatment (Lee et al. 2010). Prostatic artery embolization seems to be a promising therapeutic option even in this collective, as we observed improvement for Wassermann patterns 3–5 and modified BPH pattern 3 (pedunculated). Abt et al. stated in their publication that prostatic anatomy is the most important factor to predict clinical outcome (Abt et al. 2018). There is a lot of evidence in the recent literature, that especially patients with larger glands benefit from PAE (Assis et al. 2017; Franiel et al. 2018; Kisilevzky and Faintuch 2016; Maclean et al. 2018; Wang et al. 2016). Another anatomic feature is the presence of large adenomatous nodules in the prostatic central gland (AdBPH), which is also positively correlated with better clinical outcomes (Abt et al. 2018; Little et al. 2017). We could confirm both findings in our collective with significantly better reduction of IPSS and Qmax in patients with larger (> 100 ml) glands and presence of AdBPH. Multiple regression revealed initial volume of the prostate as the only significant predictor for postinterventional IPSS, underlining this feature once again. Interestingly, the relative volume loss showed no significant correlation with the relative reduction in IPSS and Qmax. This stands in line with previously published results, leading to the assumption, that the reduction of pressure on the intraprostatic urethra due to volume loss is not the only effect which leads to better flow rates after PAE (Sun et al. 2008). It seems intuitive, that the effect of bilateral embolization on volume is twice the effect of unilateral embolization (14% vs. 26%). The differences on IPSS scores are even more distinct (21% vs. 53%). Based on these findings, we draw the conclusion that bilateral embolization should be aimed implicitly even in cases with difficult anatomical and technical conditions.

Our study has several limitations. First, the retrospective single centre design needs to be discussed. Next, the number of patients is small, and the follow-up period is short. The time periods between intervention and follow-up visit were not always consistent. For clinical data, only Qmax and IPSS scores were collected and analysed while other features (e.g., quality of life, sexual function) were not taken into consideration. All interventions were performed by one interventionalist which might lead to expert bias. Our described classification system for BPH patterns lacks internal and external validation. As previously mentioned, the distribution of the Wasserman types was imbalanced and the classification into different subtypes was prone to selection bias. We did not calculate interobserver agreement as there were only two radiologists reading the examinations. Furthermore, as data from cystoscopy was not regularly available in all patients, we did not conduct a correlation with the MR classification for both Wassermann and the modified classification.

## Conclusion

In conclusion, our data suggests that patients with LUTS benefit from PAE regardless the macroscopic BPH pattern. Patients with median lobe dominant appearance (retrourethral) showed equivalent response to embolization compared to preurethral dominant or biurethral morphology. Higher prostate volume or presence of AdBPH are correlated with an outcome improvement after PAE and are better predictors for the effects of PAE.

## Data Availability

Data available on request from the authors.

## Abbreviations

BPH: Benign prostatic hyperplasia
PAE: Prostatic artery embolization
MRI: Magnetic resonance imaging
AdBPH: Adenomatous-dominant benign prostatic hyperplasia
LUTS: Lower urinary tract symptoms
IPSS: International Prostate Symptom Score
Qmax: Maximum uroflow rate
TZ: Transition zone
IPP: Intraprostatic protrusion
PUA: Prostatic urethral angle
PIRADS: Prostate Imaging Reporting and Data System
MRA: Magnetic resonance angiography
CBCT: Cone beam computed tomography
TURP: Transurethral resection of the prostate
PA: Prostatic artery
